# Safety and Clinical Efficacy of Mesenchymal Stem Cell Treatment in Traumatic Spinal Cord Injury, Multiple Sclerosis and Ischemic Stroke – A Systematic Review and Meta-Analysis

**DOI:** 10.3389/fneur.2022.891514

**Published:** 2022-05-30

**Authors:** Christopher Elnan Kvistad, Torbjørn Kråkenes, Cecilie Gjerde, Kamal Mustafa, Tiina Rekand, Lars Bø

**Affiliations:** ^1^Department of Neurology, Haukeland University Hospital, Bergen, Norway; ^2^Tissue Engineering Group, Department of Clinical Dentistry, University of Bergen, Bergen, Norway; ^3^Institute for Neuroscience and Physiology, University of Gothenburg, Gothenburg, Sweden; ^4^Department of Clinical Medicine, University of Bergen, Bergen, Norway

**Keywords:** ischemic stroke, mesenchymal stem cells, multiple sclerosis, regenerative medicine, traumatic spinal cord injuries

## Abstract

**Background:**

Mesenchymal stem cells (MSCs) is an attractive candidate in regenerative research and clinical trials have assessed their therapeutic potential in different neurological conditions with disparate etiologies. In this systematic review, we aimed to assess safety and clinical effect of MSC treatment in traumatic spinal cord injury (TSCI), multiple sclerosis (MS) and ischemic stroke (IS).

**Methods:**

A systematic search was performed 2021-12-10 in MEDLINE, EMBASE, Web of Science and Cochrane where clinical studies assessing MSC treatment in TSCI, MS or IS were included. Studies without control group were excluded for efficacy analysis, but included in the safety analysis. For efficacy, AIS score, EDSS score and mRS were used as clinical endpoints and assessed in a meta-analysis using the random effects model.

**Findings:**

Of 5,548 identified records, 54 studies were included. Twenty-six studies assessed MSC treatment in TSCI, 14 in MS and nine in IS, of which seven, seven and five studies were controlled, respectively. There were seven serious adverse events (SAEs), of which four were related to the surgical procedure and included one death due to complications following the implantation of MSCs. Three SAEs were considered directly related to the MSC treatment and all these had a transient course. In TSCI, a meta-analysis showed no difference in conversion from AIS A to C and a trend toward more patients treated with MSCs improving from AIS A to B as compared to controls (*p* = 0.05). A subgroup analysis performed per protocol, showed more MSC treated patients improving from AIS A to C in studies including patients within 8 weeks after injury (*p* = 0.04). In MS and IS, there were no significant differences in clinical outcomes between MSC treated patients and controls as measured by EDSS and mRS, respectively.

**Interpretation:**

MSC-treatment is safe in patients with TSCI, MS and IS, although surgical implantation of MSC led to one fatal outcome in TSCI. There was no clear clinical benefit of MSC treatment, but this is not necessarily a proof of inefficacy due to the low number of controlled studies. Future studies assessing efficacy of MSC treatment should aim to do this in randomized, controlled studies.

## Introduction

For neurological diseases affecting the central nervous system (CNS), there are no available therapies that may repair and thereby reverse neurological disability. So far, this has been the common denominator in CNS injury, regardless the cause.

Mesenchymal stem cells (MSCs), also known as mesenchymal stromal cells, are heterogeneous cells with self-renewal potential and multipotent properties that can be found in all postnatal tissues (1). MSCs do not have a unique cell marker, but are defined according to international guidelines by the presence and absence of different cell surface proteins and tri-lineage differentiation potential *in vitro* (2).

Recent studies have highlighted the systemic role of MSCs in tissue repair (3–5). In this setting, MSCs have been shown to possess regenerative capabilities, also for conditions affecting the CNS. Animal studies have revealed that MSCs can migrate toward sites of injury (6) and promote repair of myelin and neurons, thus leading to improved functional outcomes in models of central nervous diseases (7, 8). This effect is likely mediated through different mechanisms, such as the paracrine stimulation of endogenous progenitor- and stem cells through the MSC secretome (9), mitochondria donations (10), immunomodulation (11) and transdifferentiation toward neural cell lines (12).

MSCs can be obtained from different tissues, such as bone marrow (BM), adipose tissue and umbilical cord, and expanded *ex vivo*. The use of autologous or allogeneic MSCs represent no ethical concerns as compared to other stem cell therapies based on embryonal or fetal stem cells. This, along with the promising results from animal studies, have made MSCs an attractive candidate for regenerative human studies.

Numerous studies have been performed the last years assessing MSC treatment in neurological conditions. As injury to the human CNS may be caused by different mechanisms, an important question is whether MSC treatment is safe and whether it possesses a neuroregenerative effect across separate etiologies. In this systematic review and meta-analysis, we aimed to assess safety and clinical effect of MSC treatment in traumatic spinal cord injury (TSCI), multiple sclerosis (MS) and ischemic stroke (IS).

## Materials and Methods

### Protocol and Registration

This systematic review and meta-analysis was performed in accordance with the PRISMA guidelines (13). The protocol was completed before the search and registered at The National Institute for Health Research with ID CRD42021285638.

### Eligibility Criteria

Clinical studies including patients with TSCI, MS or IS treated with MSCs were included. Follow-up studies, case reports and studies without defined inclusion and exclusion criteria were excluded. Studies without control group were excluded for the efficacy analysis, but included in the safety analysis. Details concerning eligibility criteria are listed in the Supplementary Material 1. In the protocol, inclusion criteria were originally restricted to papers using the English language. This criteria was subsequently removed as a number of eligible papers were published in Chinese, and not including these could represent a bias. Therefore, papers in all languages could be included in the analysis.

### Search and Study Extraction

Studies were identified by searching the electronic databases MEDLINE (Ovid), EMBASE (Ovid), Web of Science and Cochrane Library. Variants of subject headings and free-text terms of “Mesenchymal stem cell transplantation” were applied in combination with different terms of traumatic spinal cord injury, multiple sclerosis and ischemic stroke. The complete search strings are shown in the Supplementary Material 2. The searches were performed on December 10th, 2021.

Eligibility assessment was performed in a two-step screening process. After removal of duplicates, the first screening was conducted by assessment of title and abstract. The cause for exclusion was recorded. The first screening was performed by one reviewer (CEK) in a standardized manner. The remaining studies were read in full text in a second screening. This step was performed non-independently by two unblinded reviewers (CEK/LB). Disagreements between reviewers were resolved by consensus. Data extraction was performed by using a pre-developed data extraction sheet. The following information was extracted: (1) study identity; (2) condition and its characteristics; (3) study design; (4) number of patients in treatment and control groups; (5) details concerning mesenchymal stem cell treatment, including origin of cells, timing of treatment, way of administration and cell dose; (6) safety data with adverse events (AEs)/serious adverse events (SAEs); (7) efficacy data as specified in the protocol.

For safety analysis, the AEs and SAEs considered by the authors to be related to the MSC treatment or MSC administration (possibly, likely or definitely) were registered in each study. If the authors did not state the relationship between the AE/SAE and MSC treatment/administration, all AEs/SAEs in the treatment arm were included in the analysis. For efficacy outcome analysis, American Spinal Injury Association Impairment Scale (AIS) (conversion AIS type A to B-C, and mean AIS scores) were extracted for TSCI, Expanded Disability Status Scale (EDSS) scores (patients improving, remaining stable and worsening, and mean difference in EDSS score) for MS and modified Rankin Scale (mRS) (patients with mRS 0–2 and mean mRS) for IS. Data were extracted from tables and/or graphs published in either the main paper or Supplementary Material. We contacted nine authors due to missing outcome data, and received reply from one.

### Quality Assessment

Risk of bias within the controlled studies were evaluated by using “The Revised Cochrane risk-of-bias tool for randomized trials (RoB 2)” and “The Risk Of Bias In Non-randomized Studies – of Interventions (ROBINS-I) assessment tool” for randomized and non-randomized studies, respectively. One reviewer (CEK) performed the assessments and results were reviewed a second time by another reviewer (LB) before completion. The reviews were not performed in an independent manner. Disagreements were resolved by consensus.

### Statistical Analysis

Safety data was registered by type and severity, and reported in frequency per procedure. Meta-analyses for dichotomous efficacy outcomes were performed by computing relative risks and risk differences with corresponding 95% confidence intervals using the Mantel-Haenszel method in a random effects model. For ordinal data, differences in mean were calculated with corresponding 95% confidence intervals using the inverse variance method in a random effects model. The random effects model was applied based on the assumption that the different studies were estimating different, yet related, intervention effects. Intention-to-treat data from the studies were used. If studies had multiple treatment arms with different doses of MSCs and only one control arm, the arm with the highest dose showing safety, was used in the meta-analysis of efficacy as comparison to the control group. Likewise, if studies used both intravenous and intrathecal administration modes, the arm with the intrathecal administration was used in the meta-analysis as comparison to the control group. Heterogeneity was assessed by using the inconsistency index (*I*^2^). Risk of bias across studies was not assessed due to the low number of studies available for each outcome analysis. No additional analyses were performed apart from subgroup analyses as specified in the protocol. Revman 5.4.1 software (Cochrane Collaboration, Oxford, UK) was used for the analyses.

## Results

### Study Selection and Risk of Bias

**Figure 1:**
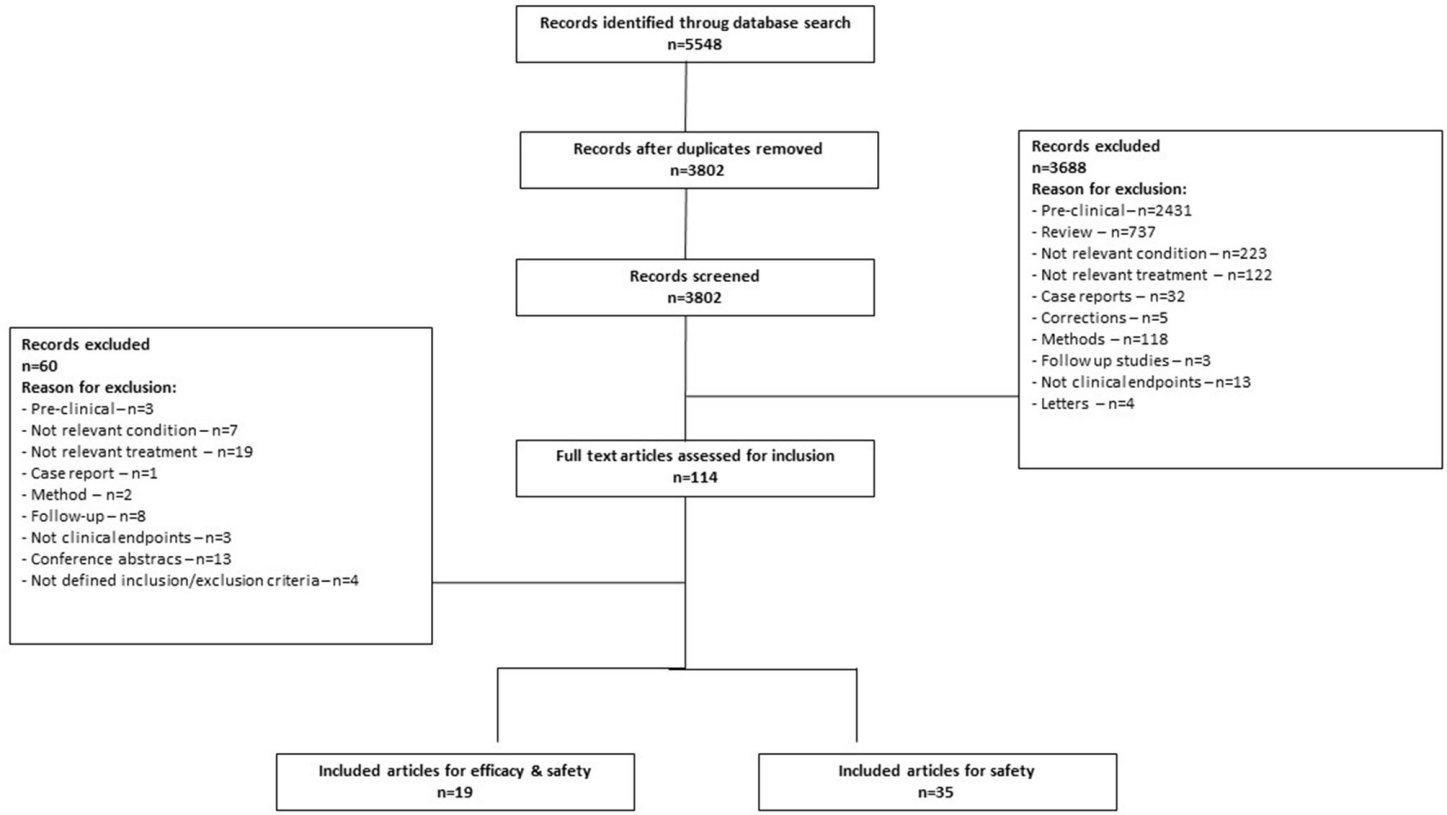
Flow diagram of the study selection process.

**Figure 2:**
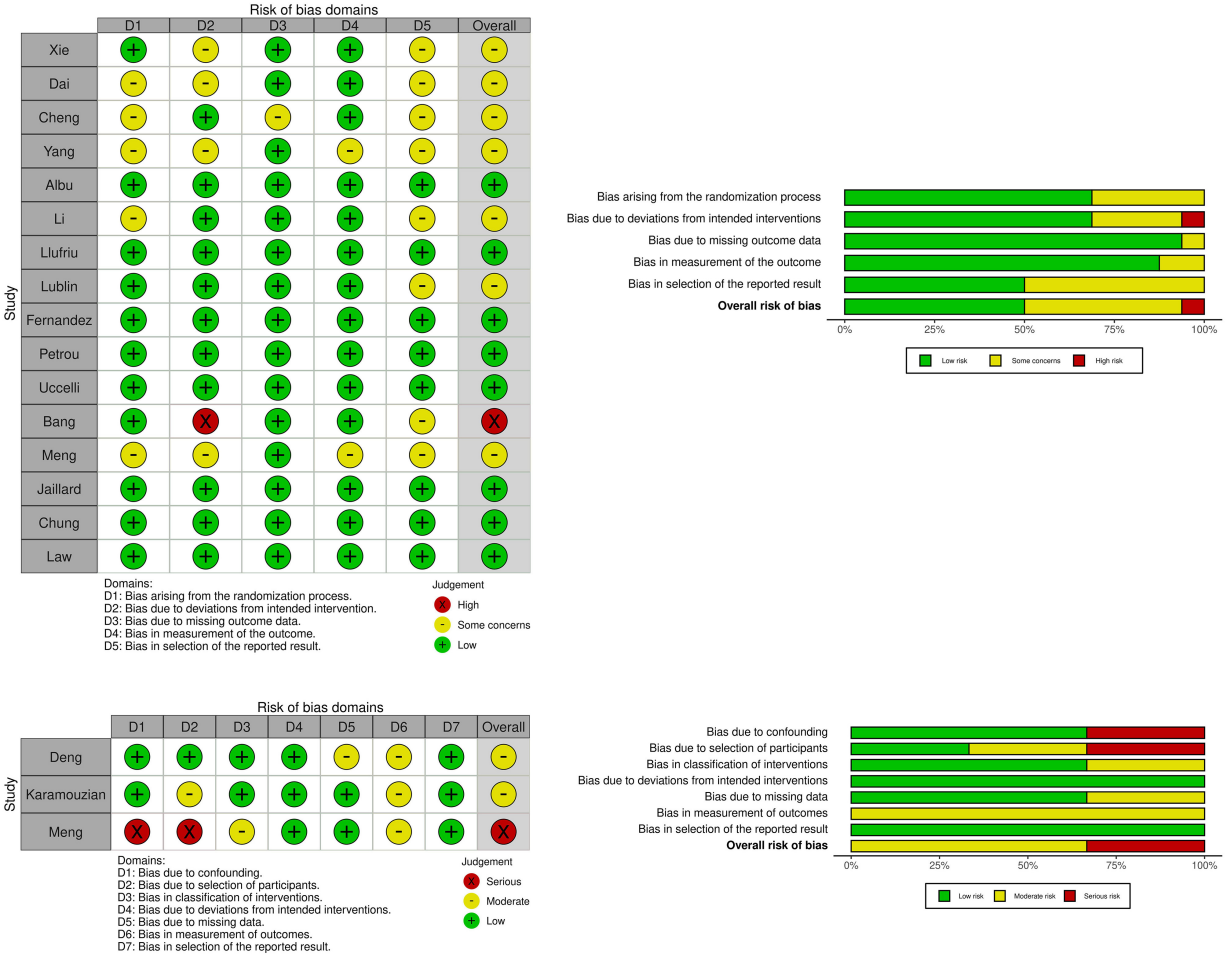
Plots showing risk of bias for controlled studies assessing efficacy of mesenchymal stem cell treatment in traumatic spinal cord injury, multiple sclerosis and ischemic stroke. Risk of bias for randomized studies (16 studies). Risk of bias for non-randomized studies (3 studies).

The search identified 5,548 records, of which 3,802 remained after duplication removal (Figure 1). After the exclusion of 3,688 records in the first screening, 114 records were assessed in full text. The second screening discarded 60 additional records due to fulfillment of various exclusion criteria. A total of 35 studies remained for safety analysis (14–48) and 19 studies for the combined efficacy and safety analysis (49–67). A summary of the risk of bias for the studies included in the efficacy and safety analysis is shown in Figure 2.

### Study Characteristics

In total, 26 of the included studies assessed TSCI (14–16, 18–32, 49–55), 19 MS (33–44, 56–62) and 9 IS (45–48, 63–67). Study characteristics are shown in Table 1.

**Table 1:** Overview over studies of mesenchymal stem cell treatment in traumatic spinal cord injury, multiple sclerosis and ischemic stroke.

**References**	**Condition and important inclusion criteria**	Timing of MSC treatment after debut of condition^*^	**Design and blinding**	**Follow-up time**	**Type of MSC & administration**	**N patients**	**N controls**
**Traumatic spinal cord injury**							
**Controlled studies**							
Xie et al. (49)	NS	1–10 months	+ Randomized ÷ Placebo ÷ Blinded	90 days	Autologous MSCs from BM IT or IV x 1	12	11
Karamouzian et al. (50)	AIS A Thoracic	2–8 weeks	÷ Randomized ÷ Placebo ÷ Blinded	20 – 23 months	Autologous MSCs from BM IT x 1	11	20
Dai et al. (51)	AIS A Cervical	≥1 year	+ Randomized ÷ Placebo + Assessor blinded	6 months	Autologous MSCs from BM IL x 1	20	20
Cheng et al. (52)	AIS A Thoracic and lumbar	NS	+ Randomized ÷ Placebo + Assessor blinded	6 months	Allogeneic MSCs from UC IL x 1	10	14 (rehabilitation group)
Deng et al. (53)	AIS A Cervical	≤ 21 days	÷ Randomized ÷ Placebo ÷ Blinded	12 months	Allogeneic MSCs from UC IL x 1 with collagen scaffold	20	20
Yang et al. (54)	NS	≥1 month	+ Randomized ÷ Placebo ÷ Blinded	1 month	Autologous MSCs from BM IL x 1	34	34
Albu et al. (55)	AIS A Thoracic	1–5 years	+ Randomized + Placebo + Double blinded + Cross-over	6 months	Allogeneic MSCs from UC IT x 1	10	10 (cross-over)
**Uncontrolled studies**							
Li et al. (14)	NS	1 week−60 months	-	1 year	Autologous MSCs from BM in combination with surreal nerve IL x 1	78	-
Pal et al. (15)	AIS NS Cervical or thoracic injury	Group – 1–6 months Group 2 – ≥6 months	-	1-3 years	Autologous MSCs from BM IT x 2-3	Group 1 – 20 patients Group 2 – 10 patients	-
Jeon et al. (16)	AIS NS Cervical	≥1 month	-	6-11 months	Autologous MSCs from BM IL x 1 & IT x 2	10	-
Bhanot et al. (17)	AIS A Level of injury NS	≥8 weeks	-	6-38 months	Autologous MSCs from BM IL x 1 and IT x 3	13	-
Yazdani et al. (18)	AIS A Level of injury NS	≥1 year	-	26-43 months	Autologous MSCs from BM and schwann cells IL x 1	8	-
Medonca et al. (19)	AIS A Thoracic or lumbar	≥6 months	-	6 months	Autologous MSCs from BM IL x 1	14	-
Oh et al. (20)	AIS B Cervical injury	≥1 year	-	6 months	Autologous MSCs from BM IL x 1	16	-
Oraee-Yazdani et al. (21)	NS	≥1 year	-	Mean 30 months	Autologous MSCs from BM and schwann cells IT x 1 1 mill MSCs	6	-
Satti et al. (22)	AIS A Thoracic injury	Group 1-2 weeks−6 months Group 2 - >6 months	-	269 – 826 days	Autologous MSCs from BM IT x 2-3	Group 1 – 3 patients Group 2 – 6 patients	-
Thakkar et al. (23)	NS	≥12 months	-	Mean 3 years	Autologous MSCs from adipose tissue and autologous hematopetic stem cells IT x 1	10	-
Vaquero et al. (24)	AIS A Level of injury NS	≥6 months	-	12 months	Autologous MSCs from BM ILx 1 and IT x 1	12	-
Larocca et al. (25)	AIS A Thoracic	≥1 year	-	6 months	Autologous MSCs from BM IL x 1	5	-
Vaquero et al. (26)	Incomplete injury Level of injury NS	≥12 months	-	12 months	Autologous MSCs from BM IT x 4	10	-
Zhao et al. (27)	AIS A Cervical or thoracic injury	≥2 months	-	1 year	Allogeneic MSCs from umbilical cord + collagen scaffold IL x 1	8	-
Vaquero et al. (28)	AIS A–D Level of injury NS	≥6 months	-	10 months	Autologous MSCs from BM IT x 3	11	-
Vaquero et al. (29)	AIS A-D Level of injury NS	≥6 months	-	6 months	Autologous MSCs from BM IL x 1	6	-
Oraee-Yazdani et al. (30)	AIS A Level of injury NS	3–12 months	-	12 months	Autologous MSCs from BM and schwann cells IT x 1	11	-
Yang et al. (31)	AIS A-D Level of injury NS	≥2 months	-	12 months	Allogeneic MSCs from UC IT x 4	102	-
Zamani et al. (32)	AIS A Thoracic injury	≥6 months	-	2 years	Autologous MSCs from BM and olfactory ensheating cells IL x 1	3	-
**Multiple sclerosis**							
**Controlled studies**							
Li et al. (56)	RRMS/SPMS EDSS 4-8 Treatment failure NR	≥2 years	+ Randomized ÷ Placebo ÷ Blinded	12 months	Allogeneic MSCs from UC in combination with methylprednisolone IV x 3	13	10
Llufriu et al. (57)	RRMS EDSS 3 – 6.5 Treatment failure	2–10 years	+ Randomized + Placebo + Double blinded + Cross-over	6-12 months	Autologous MSCs from BM IV x 1	9	9 (cross-over)
Lublin et al. (58)	RRMS/SPMS EDSS not specified Treatment failure	≥2 years	+ Randomized + Placebo + Double blinded	6-12 months	Allogeneic, placenta-derived MSCs IV x 1	12–6 low dose−6 high dose	4
Meng et al. (59)	MS type and EDSS not specified Treatment failure	NS	÷ Randomized ÷ Placebo ÷ Blinded	1 – 3 years	Allogeneic MSCs from UC IV x 7	2	1
Fernandez, et al. (60)	SPMS EDSS 5.5 – 9 Treatment failure	NS	+ Randomized + Placebo + Double blinded	12 months	Autologous adipose-derived MSCs IV x 1	23–11 low dose −12 high dose	11
Petrou et al. (61)	SPMS/PPMS EDSS 3 – 6.5 Treatment failure	≥3 years	+ Randomized + Placebo + Double blinded + Cross-over	6-12 months	Autologous MSCs from BM IL and IV x 1–2	16 IT & 16 IV	16 (cross-over)
Uccelli et al. (62)	RRMS/SPMS/PPMS EDSS 2.5-6.5 Treatment failure NR	2–15 years	+ Randomized + Placebo + Double blinded + Cross-over	24 – 48 weeks	Autologous MSCs from BM IV x 1	144	144 (cross-over)
**Uncontrolled studies**							
Bonab et al. (33)	Type MS NS EDSS ≤ 6 Treatment failure	NS	-	12 months	Autologous MSCs from BM IT x 1–2	10	-
Yamout et al. (34)	MS type NS EDSS 4 – 7.5 Treatment failure	NS	-	12 months	Autologous MSCs from BM IT x 1	10	-
Bonab et al. (35)	SPMS/PPMS EDSS 3.5 – 7 Treatment failure	2–15 years	-	12 months	Autologous MSCs from BM IT x 1	25	-
Connick et al. (36)	MS type not specified EDSS 2 – 6.5 Treatment failure NR	NS	-	6 months	Autologous MSCs from BM IV x 1	10	-
Odinak et al. (37)	MS type and EDSS NS Treatment failure	NS	-	12 months	Autologous MSCs from BM IV x 4–8	8	-
Harris et al. (38)	SPMS/PPMS EDSS ≥3 Treatment failure NS	NS	-	Mean 7.4 years	Autologous MSCs from BM (differentiated in neural direction) IT x 2–5	6	-
Dahbour et al. (39)	MS type & EDSS NS Treatment failure	NS	-	12 months	Autologous MSCs from BM IT x 2,	10	-
Cohen et al. (40)	RRMS/SPMS EDSS 3 – 6.5 Treatment failure NR	NS	-	6 months	Autologous MSCs from BM IV x 1	24	-
Harris et al. (41)	SPMS/PPMS EDSS ≥3 Treatment failure NS	NS	-	12 months	Autologous MSCs from BM (differentiated in neural direction) IT x 3	20	-
Riordan et al. (42)	MS type NS EDSS 2 – 7 Treatment failure NR	NS	-	12 months	Allogeneic MSCs from umbilical cord IV x 7	20	-
Sahraian et al. (43)	RRMS/SPMS EDSS ≤ 5.5 Treatment failure	2–15 years	-	2 years	Autologous MSCs from BM IT x 1–2	4	-
Iacobeus et al. (44)	RRMS/SPMS/PPMS EDSS 3-7 Treatment failure	2–20 years	-	48 weeks	Autologous MSCs from BM IV x 1	7	-
**Ischemic stroke**							
**Controlled studies**							
Bang et al. (63)	MCA-area NIHSS ≥7 Age 30-75	≥7 days	+ Randomized ÷ Placebo + Assessor blinded	12 months	Autologous MSCs from BM IV x 1	5	25
Meng et al. (64)	Area, NIHSS, age NS	≤ 6 weeks	+ Randomized ÷ Placebo ÷ Blinded	6 months	Autologous MSCs from BM IV x 1	30	30
Jaillard et al. (65)	Carotid area NIHSS ≥7 Age 18-70	≤ 2 weeks	+ Randomized ÷ Placebo + Assessor blinded	24 months	Autologous MSCs from BM IV x 1	16	15
Chung et al. (66)	MCA area NIHSS 6-21 Age 30-75	≤ 90 days	+ Randomized ÷ Placebo + Assessor blinded	3 months	Autologous MSCs from BM IV x 1	39	15
Law et al. (67)	MCA area NIHSS 10 – 35 Age 30 - 75	≤ 2 weeks	+ Randomized ÷ Placebo + Assessor blinded	12 months	Autologous MSCs from BM IV administration x 1	9	8
**Unontrolled studies**							
Honmou et al. (45)	Supratentorial area NIHSS NS Age 20 - 75	≤ 6 months	-	12 months	Autologous MSCs from BM IV x 1 60–160 mill cells	12	-
Qiao et al. (46)	MCA and/or ACA area NIHSS and age NS	NS	-	2 years	Allogeneic MSCs from UC and allogeneic neural stem cells from fetal brain. IV and IT	8	-
Steinberg et al. (47)	MCA area NIHSS ≥7 points Age 18 - 75 years	6–60 months	-	12 months	Allogeneic MSCs from BM IL x 1	18	-
Levy et al. (48)	Area NS NIHSS ≥6 points Age ≥18 years	≥6 months	-	12 months	Allogeneic MSCs from BM Intravenous administration x 1	Phase 1: 15 Phase 2: 21	-

*MSC, mesenchymal stem cells; AIS, American Spinal Injury Association Impairment Scale; EDSS, Expanded Disability Status Scale; NIHSS, National Institute of Health Stroke Scale; mRS, Modified Rankin Scale; NS, not specified; NR, not required; RRMS, relapsing-remitting multiple sclerosis; SPMS, secondary progressive multiple sclerosis; PPMS, primary progressive multiple sclerosis; MCA, middle cerebral artery*.

* *According to inclusion criteria*.

#### Traumatic Spinal Cord Injury

Of the 26 included studies, seven were controlled (49–55), of which five were randomized (49, 51, 52, 54, 55), one double-blinded (55) and two assessor-blinded (51, 52). Fourteen studies included patients with isolated AIS A (17–19, 22, 24, 25, 27, 30, 32, 50–53, 55), one isolated AIS B (20) whereas 11 studies included all AIS classifications or did not specify this in the inclusion criteria (14–16, 21, 23, 26, 28, 29, 31, 49, 54). Four studies included only patients with cervical injury (16, 20, 51, 53) and five included only patients with thoracic injury (22, 25, 32, 50, 55). The remaining studies included injuries in several segments of the spinal cord or did not specify this (14, 15, 17–19, 21, 23, 24, 27–31, 49, 52, 54). According to inclusion criteria, MSC treatment was administered within the first 3 weeks after injury in one study (53), within 2–8 weeks in one study (50) and after 6 months or more in 13 studies (18–21, 24–26, 28, 29, 32, 51, 55). Twelve studies used intralesional administration via surgery or guided injections (14, 18–20, 25, 27, 29, 32, 51–54) and ten studies administered the cells intrathecally *via* lumbar puncture (15, 21–23, 26, 28, 30, 31, 50, 55), whereas four used combinations of different administration methods (16, 17, 24, 49).

#### Multiple Sclerosis

A total of 19 studies were included (33–44, 56–62), of which six were randomized (56–58, 60–62) and five double-blinded (57, 58, 60–62). Five studies only included patients with progressive MS (35, 38, 41, 60, 61) and 12 studies had failure to standard disease modifying treatment as an inclusion criteria (33–35, 37, 39, 43, 44, 57–61). In 11 studies, the stem cells were given intravenously (36, 37, 40, 42, 44, 56–60, 62), in seven intrathecally (33–35, 38, 39, 41, 43) and in one study both intravenously and intrathecally (61). The follow-up period varied between 6 months and 7 years.

#### Ischemic Stroke

Nine studies were included (45–48, 63–67), of which five were controlled (63–67) and five assessor-blinded (63, 65–67). Six studies included only patients with moderate or severe stroke (47, 48, 63, 65–67), whereas this was not specified in three studies (45, 46, 64). The stem cells were administered intravenously in all studies except for one where the stem cells were also were injected intrathecally (46) and one study that used local administration (47). Patients were treated within 2 weeks after stroke onset in two studies (65, 67), whereas two studies only included patients with chronic stroke, surpassing 6 months after onset (47, 48). The follow-up time varied between 6 and 24 months.

### Safety Analysis

In 1,044 patients receiving 1,810 transplantations via either intravenous, intrathecal or intralesional administration routes, a total of 845 AEs were reported. There were 429 (70.8%) AEs for patients treated intravenously, 248 (30.7%) intrathecally, 85 (39.7%) intralesionally and 72 (39.8%) with different combinations of administration routes (in 11 patients route of administration was not specified). Of the seven reported SAEs, two received MSCs intravenously, two intrathecally and three intralesionally. Safety data are shown in Supplementary Table 3.

In TSCI, 479 patients received 713 intrathecal and 231 intralesional treatments. One SAE was reported and this was a patient who died due to complications after surgery where the MSCs were implanted (14). Fever (8%) and headache (3%) were the most common AE, irrespective of administration mode.

In patients treated with intrathecal administration, the most frequent AE per procedure was fever (9%) and headache (4%) whereas paresthesia (4%) and neuropathic pain (3%) were among the most frequent reported events in patients treated with intralesional administration.

In MS, 394 patients were treated with in total 491 intravenous and 186 intrathecal injections. Three SAEs were considered related to treatment; one anaphylactic reaction (58), one infection (62) and one transient encephalopathy with epileptic seizures (34). All these reactions had a transient course. Of the specific AEs, headache (14%) and injection site symptoms (4%) were most frequent. In patients receiving the MSC intravenously, headache (6%) and fatigue (5%) were the most commonly reported AE, and headache (32%) and fever (12%) when injected intrathecally.

A total of 171 patients with ischemic stroke were treated, of which 159 received MSC intravenously, 12 intrathecally and 18 via intralesional implantation. There were three serious adverse events; one epileptic seizure, one subdural hematoma, one pneumonia (47). All occurred in a study where MSCs were implanted in the lesion site and all were considered related to the surgical procedure. In total, headache (10%) and fever (6%) were most frequently reported as AE, irrespective of administration mode. For patients receiving only intravenous injections, fever (3%) and urinary infection (3%) were most common.

### Efficacy Analysis

**Figure 3:**
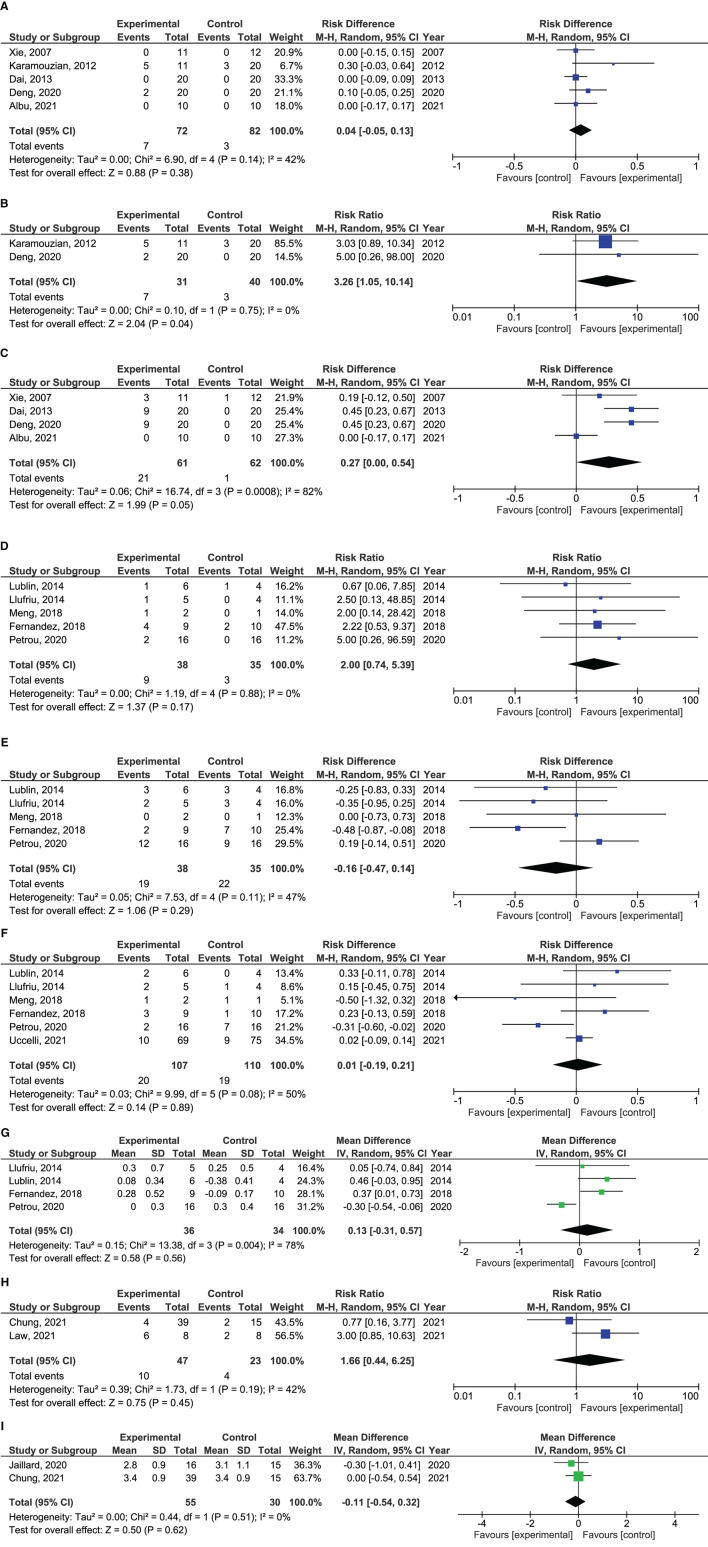
Forrest plots showing effect size of different outcomes. **(A)** Risk difference of improvement from ASIA A to ASIA C in patients with traumatic spinal cord injury treated with mesenchymal stem cells. **(B)** Risk difference of improvement from ASIA A to ASIA C in patients with traumatic spinal cord injury treated with mesenchymal stem cells within 8 weeks after injury. **(C)** Risk difference in improvement from ASIA A to ASIA B in patients with traumatic spinal cord injury treated with mesenchymal stem cells. **(D)** Risk ratio of EDSS improvement in patients with multiple sclerosis treated with mesenchymal stem cells. **(E)** Risk difference in remaining stable in EDSS in patients with multiple sclerosis treated with mesenchymal stem cells. **(F)** Risk difference in EDSS worsening in patients with multiple sclerosis treated with mesenchymal stem cells. **(G)** Mean difference in ΔEDSS scores in patients with multiple sclerosis treated with mesenchymal stem cells. **(H)** Risk ratio of patients with mRS 0-2 in patients with ischemic stroke treated with mesenchymal stem cells. **(I)** Mean difference in mRS scores in patients with ischemic stroke treated with mesenchymal stem cells.

A total of 679 patients (236 SCI, 261 MS and 182 IS) were included in the combined efficacy and safety analyses (Table 1), of which 472 patients (154 SCI, 217 MS and 101 IS) reported clinical data that enabled them to enter one or more of the pre-specified meta-analyses. The forest plots are shown in Figures 3A–I. Due to the low number of included studies, a majority of subgroup analyses specified in the protocol could not be performed. As an adjustment from protocol, worsening in EDSS was also included in the analysis.

#### Traumatic Spinal Cord Injury

Five studies reported proportion of patients with improvement from AIS classification A to C (49–51, 53, 55) and four from A to B (49, 51, 53, 55). Total AIS scores were not assessed as only one of the studies reported this (51). In the pooled analysis, there were no differences in proportion of patients converting from AIS A to C (risk diff: 0.04; 95% CI −0.05**–**0.13; *p* = 0.38) between MSC treated and controls. There was a trend toward more patients treated with MSCs improving from AIS A to B (risk diff: 0.27; 95% CI 0.00**–**0.54; *p* = 0.05). This analysis had a high heterogeneity with *I*^2^ = 82%. Exploration of heterogeneity identified one study that, in contrast to the other studies, did not report any improvement from AIS A to B in any patients (55). This was the only randomized, placebo-controlled study in the analyses, and the study was therefore not removed. A subgroup analysis performed per protocol, showed more MSC treated patients improving from AIS A to C as compared to controls when only studies including patients within 8 weeks after injury were analyzed (RR: 3.26; 95% CI 1.05**–**10.14; *p* = 0.04) (50, 53).

#### Multiple Sclerosis

Five studies reported proportion of patients with improvement and stabilization in EDSS (57–61), and six reported worsening (57, 58, 60–62). Four studies reported mean difference in EDSS after treatment (57, 58, 60, 61). There were no significant differences in rates of EDSS improvement (RR 2.00; 95% CI 0.74**–**5.39; *p* = 0.17), stabilization (Risk diff: −0.16; 95% CI −0.47**–**0.14; *p* = 0.29) or worsening (risk diff: 0.01; 95% CI −0.19**–**0.21; *p* = 0.89) between patients receiving MSCs and controls. Also, no difference was found in ΔEDSS scores between treatment groups (mean diff: 0.13; 95% CI −0.31**–**0.57; *p* = 0.56). This analysis had a high heterogeneity with *I*^2^ = 78%, which was caused by one study (61). This was the only study where MSCs were administered intrathecally and the study was not removed.

#### Ischemic Stroke

Two studies reported on proportion of patients with mRS 0**–**2 (66, 67) and total mRS scores (65, 66). There were no differences between MSC treatment vs. controls in rates of mRS 0**–**2 (RR 1.66; 95% CI 0.44**–**6.25; *p* = 0.45), or mean differences in total mRS (mean diff: −0.11; 95% CI −0.54**–**0.32; *p* = 0.62) after treatment.

## Discussion

Our systematic review showed that MSC treatment is reasonably safe and well-tolerated in TSCI, MS and IS. Low grade fever and headache were the most frequent reported adverse events. In the largest study, adverse events were registered in organ classes and no differences in safety parameters were reported between treatment and control group (62).

There were seven serious adverse events, of which three were considered directly related to the stem cell treatment; one anaphylactic reaction, one infection and one transient encephalopathy. A Chinese study reported a complication after the surgical procedure that led to the death of one patient with TSCI where MSCs in combination with sural nerve tissue was implanted into the injured spinal cord (14). This was the only serious adverse event that resulted in death. Of the events considered directly related to the treatment with MSCs, the transient encephalopathy may be regarded as the most serious. This patient had MS and received a high number of cells (100 million MSCs) intrathecally 50/50% *via* lumbar and intracisternal puncture (34). The patient developed epileptic seizures few days after transplantation, which required hospitalization and intravenous valproate. Reportedly, the patient recovered without significant sequelae. Of notice, another study reported two cases of iatrogenic meningitis as adverse events in patients with MS after intrathecal injection of MSCs (35). There were no abnormalities in CSF and microbiological studies were negative. They received antibiotics for 14 days and were discharged without sequelae. Our findings concerning safety of MSC treatment are in harmony with another review that assessed MSC treatment in a spectrum of different medical conditions and found no risk of serious complications such as tumorigenicity or toxicity (68).

In the meta-analysis of clinical efficacy, there was no overall motoric effect of MSC treatment that enabled the re-classification of AIS A to C. In our view, an ability to regain motoric function below the level of injury in patients with complete injuries would not only be the most important clinical benefit from a patient point of view, but also a clear indication of clinical efficacy. A possible effect was, however, noted in a subgroup analysis where only patients treated within 8 weeks after injury were included. This may suggest that the optimal timepoint of MSC treatment is within the first weeks after TSCI, which seems biologically reasonable in the sense that the MSCs in this time period may have better access to the injured nervous tissue in the absence of scar tissue and mature gliosis. The finding must, however, be interpreted with caution as only two studies with 71 patients in total were included in the analysis (50, 53). Also, more patients transformed from AIS A to AIS B in the MSC arm. This result should also be interpreted cautiously due to a considerable heterogeneity that was caused by the neutral results of the only double-blinded, placebo-controlled study in the analysis. Skin sensation is a more subjective parameter than motor abilities, and may thus be more prone to bias in studies where the patients are unblinded. With this backdrop, it is noteworthy that the only patient blinded trial in this analysis did not show any effect of MSC-treatment on transformation from AIS A to B (55). This trial included only patients with chronic TSCI.

In the meta-analysis of clinical efficacy in MS patients, there were no apparent clinical benefits of MSC treatment as compared to controls in either improving, stabilizing or preventing worsening in EDSS. The neutral findings are in concordance with a recently published large, randomized study that included 148 patients and failed to show an effect of intravenous MSC treatment in disease activity (62). A meta-analysis including both controlled and uncontrolled MS-studies have suggested that intrathecal administration of MSCs may be more efficacious than the intravenous route (69). This may also seem biological plausible as animal studies have shown that intravenously injected MSCs are trapped in the lungs and deposed out of the organism after short time (70, 71). These findings is also in harmony with our findings, as a majority of the included MS studies used an intravenous administration form (56–60, 62). Also the four controlled studies assessing efficacy of MSC treatment in ischemic stroke used intravenous administration (63, 65–67). Only three studies assessed mRS in a way that permitted comparison in a meta-analysis (65–67). The synthesis of these results did not show any clear clinical benefit of MSC treatment compared to the controls. These results are in concordance with a large, randomized trial that investigated safety and efficacy of intravenously administered BM-derived adult multipotent progenitor cells with similar properties as MSCs (72). The treatment was safe and well-tolerated, but could not demonstrate significant neurological improvements at 90 days after treatment.

The lack of effect in our meta-analysis is in contrast to a number of uncontrolled studies, which have reported promising results with MSC treatment in the same conditions using similar clinical endpoints (19, 23, 24, 34–39, 41, 43, 45). It is likely that there is a considerable placebo effect in studies where patients are treated with advanced medicinal treatment, such as mesenchymal stem cells. Perhaps especially in neurological conditions where no curative treatment is available. This may also provide a basis for publication bias, where positive case reports and case series are more often published than studies where no effect can be shown. Our findings, in combination with these speculations, highlight the need for future MSC studies being randomized, and if possible, blinded for the patients and assessors. Such a design has already been proposed, as the “International Mesenchymal Stem Cells Transplantation Study Group” in 2010 recommended the use of double-blinded, randomized, controlled cross-over studies for the assessment of MSC treatment in MS (73). According to clinicaltrials.gov, three MSC trials are per now recruiting patients with TSCI, four trials are recruiting MS patients and eight trials IS patients, of which two, two and six are randomized, respectively (Supplementary Table 4).

Also, in addition to applying clinical scales such as ASIA, EDSS and mRS, future trials should consider to assess more sensitive efficacy parameters in order to be able to demonstrate “proof-of-concept.”

Our systematic review has limitations. We aimed to assess the clinical effect because this is the most relevant outcome from a patient point of view. Clinical efficacy is also a parameter that is measurable and comparable across different neurological conditions. Outcomes with AIS, EDSS and mRS may however be a crude effect estimate when the number of included patients are low, such as in our analyses. In TSCI, we also only reported shifts in AIS classification due to the low number of studies reporting total AIS scores. The lack of benefit of MSC treatment in our main analysis should therefore not be interpreted as a proof of inefficacy.

Another limitation is the low number of controlled studies that entered the meta-analysis. This is mainly because only few controlled trials have been published so far and these were also slightly inconsistent in the reporting of clinical outcomes. In addition, the included studies used different administration methods and treated the patients in different time windows after the debut of the conditions. We still found it reasonable to synthetize the results in a meta-analysis, as all studies investigated the clinical effect of MSC treatment as compared to control groups.

In conclusion, our systematic review showed that MSC treatment is safe in patients with TSCI, MS and IS, although surgical implantation of MSC led to one fatal outcome in TSCI. There was no clear clinical benefit of MSC treatment in the main analyses, but this is not necessarily a proof of inefficacy due to a low number of controlled studies. Future studies assessing efficacy of MSC treatment should aim to do this in randomized, controlled studies, if possible.

## Data Availability Statement

The raw data supporting the conclusions of this article are available from the corresponding author upon reasonable request.

## Author Contributions

CK and TK designed the study. CK and LB conducted the search and extraction of data. All authors contributed to the statistical analysis plan and revised the final version of the manuscript.

## Funding

This work was funded by Helse Vest and the National Programme for Clinical Therapy Research (KLINBEFORSK).

## Conflict of Interest

The authors declare that the research was conducted in the absence of any commercial or financial relationships that could be construed as a potential conflict of interest.

## Publisher's Note

All claims expressed in this article are solely those of the authors and do not necessarily represent those of their affiliated organizations, or those of the publisher, the editors and the reviewers. Any product that may be evaluated in this article, or claim that may be made by its manufacturer, is not guaranteed or endorsed by the publisher.
