# Supplementary material for: Safety and Clinical Efficacy of Mesenchymal Stem Cell Treatment in Traumatic Spinal Cord Injury, Multiple Sclerosis and Ischemic Stroke – A Systematic Review and Meta-Analysis
Source: Front Neurol. 2022 May 30;13:891514. doi: 10.3389/fneur.2022.891514 (PMC9196044; doi:10.3389/fneur.2022.891514)
Supplement: Supplementary file 1 [file Table_1.pdf]

Supplemental material:

S1: Inclusion & exclusion criteria

Inclusion criteria:

- Clinical studies that include only patients with traumatic spinal cord injury, multiple sclerosis or ischemic stroke assessing efficacy and safety of treatment with mesenchymal stem cells

Exclusion criteria:

- Studies without control-group (only for efficacy, these studies are included for safety-analyses)
- Follow-up studies (studies assessing the same study population at a later time point than the primary article did)
- Case-series or case-reports
- Studies without defined inclusion and exclusion criteria

S2: Search strings

Medline 2021-12-10

Ovid MEDLINE(R) and Epub Ahead of Print, In-Process, In-Data-Review & Other Non-Indexed Citations, Daily and Versions(R) <1946 to December 09, 2021>

1	exp Mesenchymal Stem Cell Transplantation/	13629
2	(mesenchymal adj3 cell*).ti,ab,kf.	79954
3	1 or 2	81484
4	multiple scleros*.ti,ab,kf.	83105
5	exp Multiple Sclerosis/	64265
6	4 or 5	90947
7	exp Spinal Cord Injuries/	52077
8	Spinal cord injury.ti,ab,kf.	38746
9	Spinal cord injuries.ti,ab,kf.	6595
10	Spinal cord trauma.ti,ab,kf.	925
11	Spinal cord contusion.ti,ab,kf.	716
12	7 or 8 or 9 or 10 or 11	64955
13	ischemic stroke*.ti,ab,kf.	55532
14	ischaemic stroke*.ti,ab,kf.	8116
15	Brain ischemia*.ti,ab,kf.	5880

16	Brain ischaemia.ti,ab,kf.	531
17	Cerebral infarction*.ti,ab,kf.	16944
18	exp Ischemic Stroke/	4259
19	exp Brain Ischemia/	116582
20	13 or 14 or 15 or 16 or 17 or 18 or 19	158528
21	6 or 12 or 20	311846
22	3 and 21	2010

Embase 2021-12-10

Embase <1974 to 2021 Week 48>

1	exp Mesenchymal Stem Cell Transplantation/	13352
2	(mesenchymal adj3 cell*).ti,ab,kf.	110642
3	1 or 2	112799
4	multiple scleros*.ti,ab,kf.	129446
5	exp Multiple Sclerosis/	141551
6	4 or 5	155539
7	exp spinal cord injury/	81890
8	Spinal cord injury.ti,ab,kf.	52106
9	Spinal cord injuries.ti,ab,kf.	9522
10	Spinal cord trauma.ti,ab,kf.	1155
11	Spinal cord contusion.ti,ab,kf.	894
12	7 or 8 or 9 or 10 or 11	90130
13	exp ischemic stroke/	8263
14	exp brain ischemia/	201782
15	ischemic stroke*.ti,ab,kf.	95541
16	ischaemic stroke*.ti,ab,kf.	13513
17	brain ischemia*.ti,ab,kf.	9075
18	brain ischaemia*.ti,ab,kf.	751

19	cerebral infarction*.ti,ab,kf.	25502
20	13 or 14 or 15 or 16 or 17 or 18 or 19	251043
21	6 or 12 or 20	489762
22	3 and 21	3549
23	limit 22 to article	1959

Cochrane 2021-12-10

ID	Search	Hits
#1	MeSH descriptor: [Mesenchymal Stem Cell Transplantation] explode all trees	241
#2	(mesenchymal NEAR/3 cell*):ti,ab,kw	1963
#3	#1 OR #2	1963
#4	MeSH descriptor: [Multiple Sclerosis] explode all trees	3791
#5	(multiple scleros*):ti,ab,kw	11255
#6	#4 OR #5	11255
#7	MeSH descriptor: [Spinal Cord Injuries] explode all trees	1841
#8	(spinal cord injury):ti,ab,kw	3494
#9	(spinal cord injuries):ti,ab,kw	2178
#10	(spinal cord trauma):ti,ab,kw	286
#11	(spinal cord contusion):ti,ab,kw	24
#12	#7 or #8 or #9 or #10 or #11	4021
#13	MeSH descriptor: [Ischemic Stroke] explode all trees	166
#14	MeSH descriptor: [Brain Ischemia] explode all trees	3876
#15	(ischemic stroke*):ti,ab,kw	16128
#16	(ischaemic stroke*):ti,ab,kw	16128
#17	(brain ischemia):ti,ab,kw	7230
#18	(brain ischaemia):ti,ab,kw	7230

#19	(cerebral infarction):ti,ab,kw	5116
#20	#13 or #14 or #15 or #16 or #17 or #18 or #19	22150
#21	#6 or #12 or #20	37136
#22	#3 and #21	181

Web of science 2021-12-10

10	#8 NOT #7 and Articles (Document Types)	1,398
9	#8 NOT #7	2,363
8	#1 AND #6	3,595
7	TS=((("animal" or "animals" or "canine*" or "dog" or "dogs" or "feline" or "hamster*" or "lamb" or "lambs" or "mice" or "monkey" or "monkeys" or "mouse" or "murine" or "pig" or "pigs" or "piglet*" or "porcine" or "primate*" or "rabbit*" or "rats" or "rat" or "rodent*" or "sheep*" or "veterinar*") NOT ("human*" or "patient*")))	4,069,994
6	#5 OR #4 OR #3 OR #2	341,554
5	TS=(cerebral infarction*)	35,509
4	TS=((ischem* OR ischaem*) near/2 (stroke* or brain))	120,210
3	TS=((spinal cord) near/2 (injur* OR trauma OR contusion))	65,720
2	TS=('multiple sclerosi*')	138,348
1	TS=(Mesenchymal near/2 cell*)	111,870

S3: Safety data with type and number of adverse- and serious adverse events reported from studies that included patients with traumatic spinal cord injury, multiple sclerosis and ischemic stroke treated with mesenchymal stem cells.

	N patients	IT*	IV*	IL*	NS*	Fever	Headache	Fatigue	Spasticity/rigidity	Dizziness/nausea	Paraesthesia/numbness	Neuropathic pain	Back pain	Other pain	Urinary infection	Respiratory infection	Other infection	Psychiatric symptoms	Abdominal symptoms	Injection site symptoms	Spinal fluid leak	Other AE	Unspecified AE	SAE**	Total all adverse events
**Spinal cord injury**																									
**Xie**	11				11	7	2				1								1						11
**Karamouzian**	11	11										8													8
**Dai**	20	20				2	1			1	2		2												8
**Cheng**	10			10								1													1
**Deng**	20			20											5	4		4	6			6			25
**Yang**	34			34		2	1						1												4
**Albu**	10	10					1			1			1												3
**Li**	78			78																				Death due to wound complication (n=1)	1
**Bhanot**	13	26		13		2			6	3		3		3											17
**Jeon**	10	20		10							1														1
**Larocca**	5			5																	1				1
**Medonca**	14			14																	1				1
**Oh**	16			16					2		6														8
**Oraee-Yazdani, 2016**	6	6																							0
**Oraee-Yazdani, 2021**	11	11					2		5		4	2										2			15
**Pal**	30	73										2													2
**Satti**	10	27					1				2														3
**Thakkar**	10	10													4										4
**Vaquero, 2016**	12	12		12		5							5	4						1		7			22
**Vaquero, 2017**	10	40				1	4						1	2											8
**Vaquero, 2018**	11	33				4						4	4												12
**Vaquero, 2018**	6	6																							0
**Yang**	102	408				54	16		6	5															81
**Yazdani**	8			8					2		2	3								2					9
**Zamani**	3			3			2		1			2													5
**Zhao**	8			8																					
**Total spinal cord injury**	**479**	**713**		**231**	**11**	**77**	**30**		**22**	**10**	**18**	**25**	**14**	**9**	**9**	**4**		**4**	**7**	**3**	**2**	**15**		**1**	**250**
**Total IT treatment**	**237**	**655**				**61**	**25**		**11**	**7**	**8**	**16**	**8**	**2**	**4**							**2**			**144**
**Total IL treatment**	**196**			**196**		**2**	**3**		**5**		**8**	**6**	**1**		**5**	**4**		**4**	**6**	**2**	**2**	**6**		**1**	**55**
**Total both IT+ IL**	**35**	**58**		**35**		**7**			**6**	**3**	**1**	**3**	**5**	**7**						**1**		**7**			**40**
**Multiple sclerosis**																									
**Li**	13		39																						0
**Llufriu**	9		9													1	3					1			5
**Lublin**	12		12				1													6				Anaphylactic reaction (n=1)	8
**Meng**	2		14			2	5			7										8					22

**Fernandez**	23		23												1	1						2			4
**Petrou**	32	32	32				19						4	1											24
**Uccelli**	144		144																				231	Infection (n=1)	232
**Bonab, 2007**	10	11					9															2[a]			11
**Bonab, 2012**	25	25				b	3			2												2			7
**Cohen**	24		24				4	2	11		2		2		10	5	3	5	3			30			77
**Connick**	10		10												1	1						2			4
**Dahbour**	15	27[c]				6	8												1	14		1			30
**Harris, 2016**	6	15				1	5																		6
**Harris, 2018**	20	60				5	18								9			1				3			36
**Lacobeus**	7		7																						0
**Odinak**	8		37					4																	4
**Riordan**	20		140				17	17			1						1		1			4			41
**Sahraian**	4	6				6	6																		12
**Yamout**	10	10											1											Transient encephalopathy (n=1)	2
**Total multiple sclerosis**	**394**	**186**	**491**			**20**	**95**	**23**	**11**	**9**	**3**		**7**	**1**	**21**	**8**	**7**	**6**	**5**	**28**		**47**	**231**	**3**	**525**
**Total IT treatment**	**90**	**154**				**18**	**49**			**2**			**1**		**9**			**1**	**1**	**14**		**6**		**1**	**104**
**Total IV treatment**	**272**		**459**			**2**	**27**	**23**	**11**	**7**	**3**		**2**		**12**	**8**	**7**	**5**	**4**	**14**		**41**	**231**	**2**	**397**
**Total both IT+ IV**	**32**	**32**	**32**				**19**						**4**	**1**											**24**
**Ischemic stroke**																									
**Bang**	5		5														1								1
**Meng**	30		30			4	3																		7
**Jaillard**	16		16											1	3	2		2				9			17
**Chung**	39		39																			2			2
**Law**	9		9																						0
**Honmou**	12		12			1				1										1					3
**Levy**	36		36												1					1					2
**Qiao**	6	12	12			7				1															8
**Steinberg**	18			18			15	2	4	4	2													Epileptic seizure (n=1); subdural hematoma (n=1); pneumonia (n=1)	30
**Total ischemic stroke**	**171**	**12**	**159**	**18**		**12**	**18**	2	4	**6**	2			**1**	**4**	**2**	**1**	**2**		**2**		**11**		**3**	**70**
**Total IV treatment**	**147**		**147**			**5**	**3**			**1**				**1**	**4**	**2**	**1**	**2**		**2**		**11**			**32**
**Total IL treatment**	**18**			**18**			**15**	**2**	**4**	**4**	**2**													**3**	**30**
**Total both IT+ IV**	**6**	**12**	**12**			**7**				**1**															**8**
**Total all conditions**	**1044**	**911**	**650**	**249**		**109**	**143**	**25**	**37**	**25**	**23**	**25**	**21**	**11**	**34**	**14**	**8**	**12**	**12**	**33**	**2**	**73**	**231**	**7**	**845**

IT: intrathecal; IV: intravenous; IL: intralesional; NS: not specified; AE: adverse events; SAE: serious adverse events

* Number of treatments

** Specified as related to treatment or procedure

a Iatrogenic meningitis

b In paper only specified as “Almost all participants”

c Second injection was condition medium

S4: Search in clinicaltrials.gov for "spinal cord injury", "multiple sclerosis" and "ischemic stroke" in combination with "mesenchymal stem cells" narrowed to "recruiting" trials (search performed April 2022)

Center (NCT-numer)	Condition and important inclusion criteria	Timing of MSC treatment after debut of condition*	Design and blinding	Primary endpoint	Follow-up time	Type of MSC & administration	N patients	Estimated study completion
**Spinal cord injury**								
**Minnesota, United States (NCT04520373)**	ASIA A/B	>1 year	+Randomized<br>÷ Placebo<br>÷ Blinded<br>+ Cross-over	ASIA	12 months	Autologous adipose-derived MSCs<br>IT x 1	40	2024
**St. John's, Antigua and Barbuda (NCT05152290)**	Spinal cord injury	NS	÷ Randomized<br>÷ Placebo<br>÷ Blinded	Safety	4 years	Allogeneic MSCs from umbilical cord<br>IT & IV x 1	20	2026
**Spain (NCT02917291)**	ASIA A/B<br>Thoracic	<120 hours	+ Randomized<br>÷ Placebo<br>+ Blinded	Safety	1 year	Allogeneic adipose-derived MSCs<br>IL x 1	48	2023
**Multiple sclerosis**								
**Bergen, Norway (NCT04749667)**	SPMS/PPMS<br>EDSS 4-7<br>Treatment failure NR	2-15 years	+ Randomized<br>+ Placebo<br>+ Double blinded<br>+ Cross-over	Neurophysiological parameters	6 months	Autologous BM-derived MSCs<br>IT x 1	18	2025
**St. John's, Antigua and Barbuda (NCT05003388)**	Multiple sclerosis	NS	÷ Randomized<br>÷ Placebo<br>÷ Blinded	Safety	4 years	Allogeneic MSCs from umbilical cord<br>IV x 1	15	2025
**Texas, United States (NCT05116540)**	RRMS<br>EDSS 3 – 6.5<br>Treatment failure NR	>6 months	+ Randomized<br>+ Placebo<br>+ Double blinded	Quality of life	1 year	Autologous adipose-derived MSCs<br>IV x 6	30	2023
**United States (NCT04956744)**	Multiple sclerosis<br>Treatment failure	NS	÷ Randomized<br>÷ Placebo<br>÷ Blinded	Safety	60 months	Allogeneic embryonic MSC<br>IV x 1	30	2027
**Ischemic stroke**								
**Shenyang, China (NCT04811651)**	Anterior circulation<br>NIHSS 6-25<br>Age 18-80	<6 months	+ Randomized<br>+ Placebo<br>+ Double blinded	mRS	90 days	Allogeneic MSCs from umbilical cord<br>IV x 1	200	2023
**Madrid, Spain (NCT04280003)**	MCA-area<br>NIHSS 8 - 20<br>Age >18	<4 days	+ Randomized<br>+ Placebo<br>+ Double blinded	Safety	24 months	Allogeneic adipose-derived MSCs<br>IV x 1	30	2023

**Hanoi, Vietnam (NCT05292625)**	NIHSS ≥5<br>Age 40 - 75	<24 months	+Randomized<br>÷ Placebo<br>÷ Blinded	Safety	12 months	Allogeneic MSCs from umbilical cord<br>IV/IT x 2	48	2023
**Taipei, Taiwan (NCT04097652)**	Hemispheric, cortical infarct<br>NIHSS 5 - 20<br>Age 20 - 80	48 – 168 hours	÷ Randomized<br>÷ Placebo<br>÷ Blinded	Safety	3 months	Allogeneic MSCs from umbilical cord<br>IV x 1	9	2023
**Taipei, Taiwan (NCT04434768)**	MCA-area<br>NIHSS 8 - 17<br>Age 20 - 80	<36 hours	÷ Randomized<br>÷ Placebo<br>÷ Blinded	Safety	24 weeks	Allogeneic MSCs from umbilical cord<br>IV x 1	14	2023
**Beijing, China (NCT04590118)**	NIHSS 6 - 20<br>Age ≥18	>6 months	+ Randomized<br>+ Placebo<br>+ Double blinded	Safety	12 months	Allogeneic MSC (source NS)<br>IV x 1	60	2023
**Beijing, China (NCT04953663)**	NIHSS 6 - 20<br>Age ≥18	>6 months	+ Randomized<br>+ Placebo<br>+ Double blinded	Safety	12 months	Allogeneic BM-derived MSC<br>IV x 1	60	2023
**Shanghai, China (NCT04093336)**	NIHSS 4 - 18<br>Age 18 - 80	<7 days	+ Randomized<br>+ Placebo<br>+ Double blinded	Safety	24 months	Allogeneic MSCs from umbilical cord<br>IV x 1	120	2024

MSC; mesenchymal stem cells, AIS; American Spinal Injury Association Impairment Scale , EDSS; Expanded Disability Status Scale, NIHSS; National Institute of Health Stroke Scale, mRS; Modified Rankin Scale, NS; not specified, NR; not required, RRMS; relapsing-remitting multiple sclerosis, SPMS; secondary progressive multiple sclerosis, PPMS; primary progressive multiple sclerosis, MCA; middle cerebral artery

*According to inclusion criteria
